# Free fatty acid receptors in the endocrine regulation of glucose metabolism: Insight from gastrointestinal-pancreatic-adipose interactions

**DOI:** 10.3389/fendo.2022.956277

**Published:** 2022-09-28

**Authors:** Yu-Feng Zhao

**Affiliations:** Institute of Basic Medical Sciences, Xi’an Medical University, Xi’an, China

**Keywords:** free fatty acid receptors, glucose metabolism, pancreatic islet cells, gastrointestinal hormones, adipose tissue

## Abstract

Glucose metabolism is primarily controlled by pancreatic hormones, with the coordinated assistance of the hormones from gastrointestine and adipose tissue. Studies have unfolded a sophisticated hormonal gastrointestinal-pancreatic-adipose interaction network, which essentially maintains glucose homeostasis in response to the changes in substrates and nutrients. Free fatty acids (FFAs) are the important substrates that are involved in glucose metabolism. FFAs are able to activate the G-protein coupled membrane receptors including GPR40, GPR120, GPR41 and GPR43, which are specifically expressed in pancreatic islet cells, enteroendocrine cells as well as adipocytes. The activation of FFA receptors regulates the secretion of hormones from pancreas, gastrointestine and adipose tissue to influence glucose metabolism. This review presents the effects of the FFA receptors on glucose metabolism *via* the hormonal gastrointestinal-pancreatic-adipose interactions and the underlying intracellular mechanisms. Furthermore, the development of therapeutic drugs targeting FFA receptors for the treatment of abnormal glucose metabolism such as type 2 diabetes mellitus is summarized.

## Introduction

Glucose homeostasis ensures continuous energy supply to all the cells of the body. It protects the body against hypoglycemic shock and hyperglycemia-induced damage to the cells such as vascular cells and neurons. In general, the uptake of glucose is intermittent while its consumption is a continual process. The fluctuation of blood glucose is well restricted to a limited range by the neuronal and hormonal regulatory molecules, which constitute a complex network to target on the organs that are critical for the intake, absorption, storage, conversion and consumption of glucose (1, 2). In this regulation network, the hormones from pancreatic islet cells locate in the central position, with the assistance of hormones from gastrointestinal enteroendocrine cells (EECs) and adipocytes (3–5).

Similarly, to glucose, free fatty acids (FFAs) are important substrates and their metabolism is entangled with glucose for energy supply. FFAs show diverse regulatory effects on glucose metabolism according to their length and saturation (6). It is well known that FFAs enter into cells for β-oxidation and generate acetyl-CoA to link glucose metabolism *via* tricarboxylic acid cycle in mitochondria. Increasing studies have demonstrated that FFAs also function as extracellular ligands to activate G protein-coupled receptors (GPCR) on the plasma membrane. GPR40, GPR120, GPR84, GPR41 and GPR43 are identified as FFA receptors, and they are differently activated by long-chain, medium-chain, and short-chain FFAs (7–9).

FFA receptors are expressed in the cells that are critical to glucose metabolism. Pancreatic islet cells, EECs and adipocytes are equipped with FFA receptors in a cell-specific manner. At present, studies have shown that FFA receptors activation regulates the endocrine function of pancreatic islet cells, EECs and adipocytes, which takes part in the regulation of glucose homeostasis (8, 10). The integrated effects of FFA receptors on glucose metabolism *via* the hormonal gastrointestinal-pancreatic-adipose (G-P-A) interactions and the underlying intracellular molecular mechanisms are summarized in this review. The drug development targeting FFA receptors for the therapy of abnormal glucose metabolism such as type 2 diabetes mellitus (T2DM) is also discussed.

## G-P-A interactions and glucose homeostasis

Insulin is secreted from islet β-cells and plays vital role in lowering blood glucose levels by acting on insulin-sensitive tissues and organs such as liver, skeletal muscles and adipose tissue. It stimulates the synthesis of glycogen and triglyceride and inhibits lipolysis to force the entry of blood glucose into cells (11). In contrast, glucagon is secreted from islet α-cells to elevate blood glucose levels by stimulating glycogenolysis, gluconeogenesis and lipolysis (12). Somatostatin (SS) and pancreatic polypeptide (PP) that are respectively secreted by islet δ-cells and γ-cells modulate insulin and glucagon secretion in a paracrine manner (13). The secretion of insulin and glucagon is primarily regulated by blood glucose, while it is also finely modulated by gastrointestinal hormones (GI hormones) and adipokines (14, 15).

GI hormones are a number of peptides that are secreted by different EECs (16). Glucagon like peptide-1 (GLP-1), cholecystokinin (CCK), gastric inhibitory peptide (GIP), ghrelin, gastrin and secretin are the well-known hormones that related to metabolism, and most of them take part in glucose metabolism by regulating insulin secretion or by acting directly on adipose tissue, liver, skeletal muscle and hypothalamus in central nervous system (CNS) (14) (17, 18). Adipokines are another group of proteins that are released by adipocytes. Among adipokines, leptin and adiponectin are well-known for their role in glucose metabolism (19). Leptin acts on CNS to inhibit appetite and stimulate sympathetic system to increase thermogenesis, and it also acts on islet β-cells to inhibit insulin secretion (20, 21). Leptin induces the loss of fat mass and the improvement of insulin sensitivity, which is beneficial to blood glucose control (22–24). However, leptin resistance occurs in obese subjects, which may contribute to the development of obesity and insulin resistance (25, 26). Adiponectin protects pancreatic islet β-cells against apoptosis and prevents islets loss after transplantation (27–29). Adiponectin also increases insulin sensitivity to improve glucose metabolism (30). The expression of leptin and adiponectin is regulated by islets hormones and GI hormones. For instance, leptin expression is increased by insulin (31), and adiponectin expression is upregulated by GLP-1 but inhibited by GIP (32–34).

**Figure 1:**
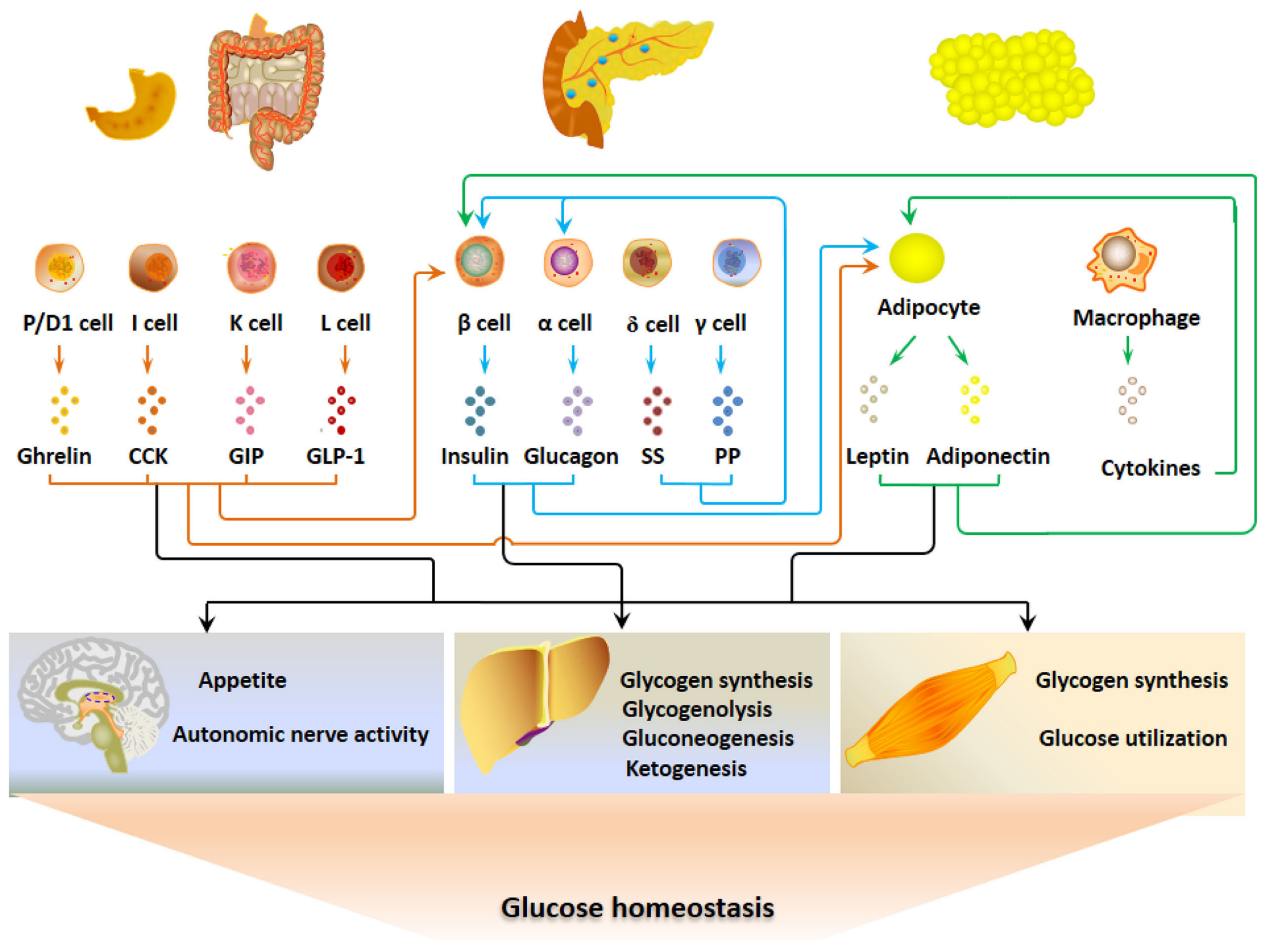
The gastrointestinal-pancreatic-adipose (G-P-A) interactions and hormonal regulation of glucose metabolism. When the blood glucose levels elevate after ingestion, insulin and certain GI hormones increase and act on the main target organs that include liver, skeletal muscle and central nervous system to lower blood glucose. Adipokines regulate glucose metabolism by altering appetite, insulin secretion, insulin sensitivity and glucose utilization, which may be a long-term mechanism for glucose metabolism.

The hormonal signals link pancreatic islet cells, EECs and adipocytes together to constitute a regulatory system for glucose metabolism (**Figure 1**). The G-P-A network responds to the fluctuation of blood glucose through a negative feedback mechanism to maintain glucose homeostasis. When the blood glucose level elevates after ingestion, insulin and some GI hormones increase to lower blood glucose by stimulating glycogen synthesis, inhibiting appetite, and reducing gluconeogenesis (35). Thus, the blood glucose is finely controlled in the normal range. Adipokines influence appetite, insulin secretion, insulin sensitivity and glucose utilization, which may be involved in the long-term mechanism for glucose metabolism.

## The effects of FFAs on glucose metabolism

Glucose and FFAs are entangled in energy metabolism, and their interaction is crucial to the maintenance of glucose homeostasis. Fatty acids can be divided into short-chain (C2-C5), medium-chain (C6-C12) and long-chain fatty acids (C14-C26). All of them regulate insulin secretion. LCFFAs are divided into saturated and unsaturated FFAs, both of which are involved in insulin secretion. In general, FFAs exhibit rapid potentiation of glucose-stimulated insulin secretion (GSIS) on the basis of elevated blood glucose levels (6, 36, 37). Meanwhile, FFAs enhance the secretion of gastrointestinal hormones such as GLP-1, CCK and GIP, which are able to promote insulin secretion (38). The acute potentiation of insulin secretion may be helpful for the control of postprandial elevation of blood glucose. However, long-term elevation of FFAs in combination with glucose damages GSIS, induces insulin resistance, and leads to the elevation of basal blood glucose (39). In addition, FFAs in long-term incubation induce lipotoxic β-cell damage and contribute to the occurrence of T2DM (40, 41).

During fasting, FFAs show the beneficial effects on glucose metabolism. LCFFAs are used as the main energy fuel and go to β-oxidation to generate energy during fasting, and they are also converted to ketone bodies for energy supply. Then blood glucose is saved, and the hypoglycemia is prevented. Blood glucose can not remain stable without the usage of LCFFA during fasting and starvation. LCFFAs are also vital for the recovery of high insulin-secreting ability of pancreatic β-cells in response to glucose after ingestion (42, 43), which is crucial to the control of glucose homeostasis after refeeding.

In summary, the influence of FFAs on glucose metabolism can be physiologically beneficial to remain glucose homeostasis at low glucose levels. On the other hand, they lead to the pathological change and the occurrence of metabolic diseases at high concentrations in accompanying high glucose.

## FFA receptors

It was previously considered that FFAs regulate glucose metabolism through intracellular metabolism. The discovery of FFA receptors that include GPR40 (FFA1), GPR120 (FFA4), GPR41 (FFA3) and GPR43 (FFA2) unveils a new mechanism of FFAs for their regulation of glucose metabolism. GPR40 and GPR120 are activated by LCFFA, while GPR43 and GPR41 are activated by SCFAs (8). Being members of the GPCR family, all the FFA receptors couple to heterotrimeric G proteins that are composed of α-subunit, β-subunit and γ-subunit (44). FFA receptors have been reported to activate multiple signaling pathways that are mediated by Gαs, Gαi/o, Gαq/11 subunits and β-arrestins (8). In a cell specific manner, GPR40 is coupled to Gαs, Gαi/o and Gαq/11 subunits, while GPR120 is coupled to Gαq/11, Gαi/o and β-arrestin2, respectively. GPR41 and GPR43 are coupled to Gαi/o, and GPR43 also couples to Gαq/11 subunit (8). The diversity of linkage between FFA receptors and G proteins enables the FFA receptor to execute flexible regulatory functions. The signaling pathways for FFA receptors have not been fully discovered, and the details of the relationship between intracellular signaling molecules and cellular responses needs to be further clarified.

It is well known that Gαs and Gαi/o affect the activity of adenylate cyclase (AC) and regulate intracellular cAMP levels and the relative signaling pathways (45). Gαq/11 is linked to phospholipase C (PLC) and activates the phosphatidylinositol signaling pathway (46). These signaling pathways regulate hormone secretion by altering the active state of many proteins including ion channels, vesicle trafficking proteins and exocytosis-related proteins. GPCR activation can recruit β-arrestins to the membrane for their binding to GPCR. β-Arrestins mediate the endocytosis of GPCR and negatively regulate GPCR signaling. Meanwhile, β-arrestins also interact with the intracellular signaling proteins (47). Mitogen-activated protein kinases (MAPK) cascade is an important signaling pathway for β-arrestin-activated intracellular signaling molecules (48).

## GPR40 and glucose metabolism

**Figure 2:**
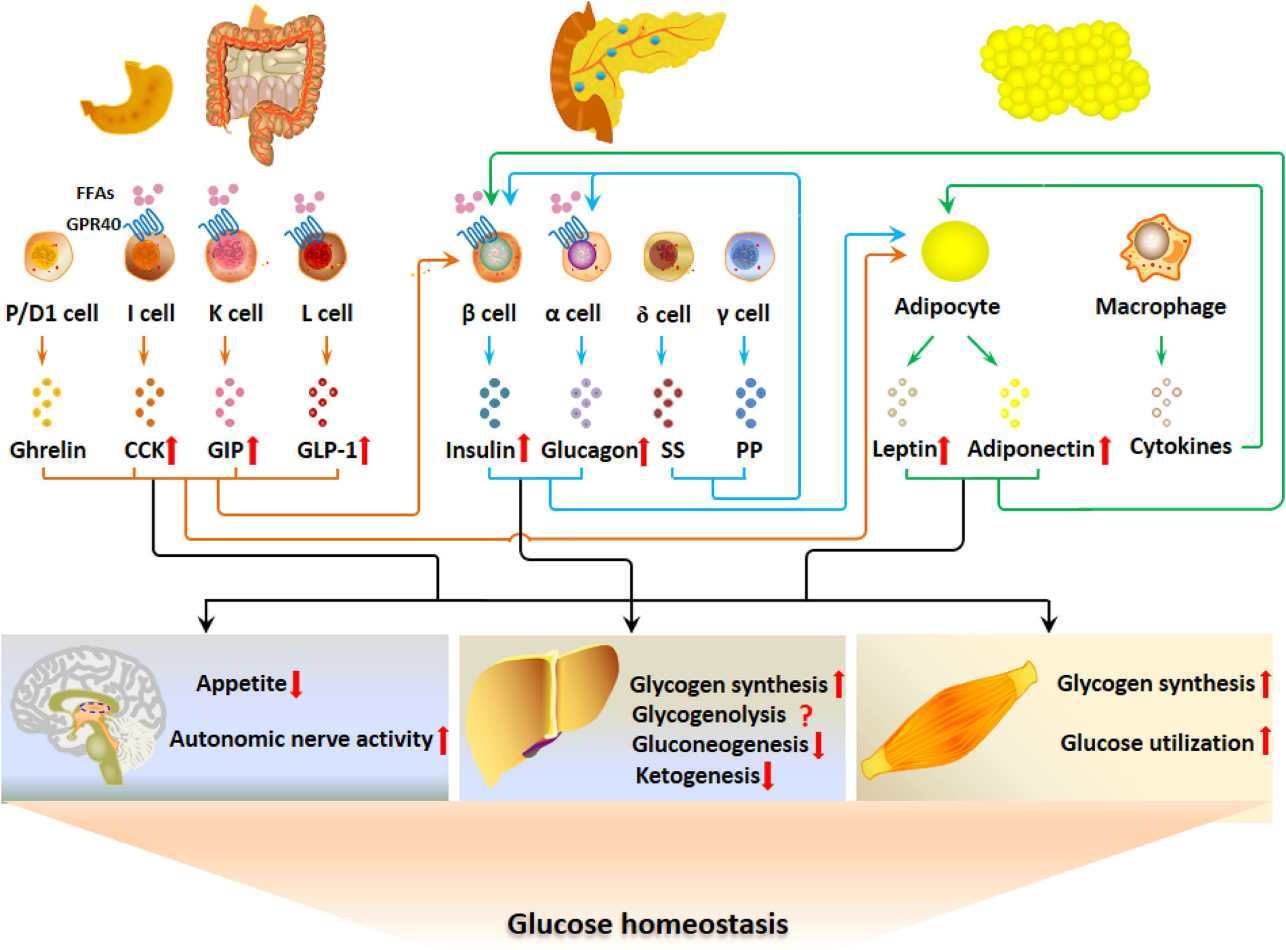
GPR40 regulates glucose metabolism *via* the G-P-A regulatory system. The activation of GPR40 by FFAs after fat ingestion potentiates the secretion of insulin, GLP-1, CCK and GIP, which contributes to the restrain of blood glucose elevation by acting on CNS, liver and skeletal muscle. Although GPR40 is not expressed in adipocytes, GPR40-potentiated insulin and GI hormones act on adipocytes to improve glucose uptake and utilization as well as adipokine secretion.

GPR40 is distributed in pancreatic islet cells and EECs. The hormonal regulation of glucose metabolism by GPR40 is summarized in **Figure 2**. GPR40 activation by FFAs after fat ingestion potentiates the secretion of insulin, GLP-1, CCK and GIP, which restrain the elevation of blood glucose by acting on CNS, liver and skeletal muscle. Although GPR40 is not expressed in adipocytes, insulin and GI hormones act on adipocytes to improve glucose uptake and utilization as well as adipokine secretion.

### GPR40 and islet hormone secretion

GPR40 was first discovered as a FFA receptor in pancreatic islet β-cells (49). GPR40 activation was reported to potentiate insulin secretion in primary cultured murine β-cells, INS-1 cells, MIN6 cells and human islets *in vitro* as well as in human and rodents *in vivo* (50–53). GPR40 knockout mice show approximately 50% reduction in FFAs-induced insulin secretion *in vivo*. It is suggested that postprandial increase of blood FFAs activates GPR40 to potentiate GSIS. The activation of PLC *via* Gαq/11 subunit and the increase in intracellular Ca^2+^ concentrations ([Ca^2+^]i) mediate GPR40-potentiated insulin secretion (51, 54). The coupling of GPR40 to Gαs subunit and the activation of AC are also suggested to mediate the effects of GPR40 agonists on ion channels activities (55).

The potentiation of insulin secretion by GPR40 activation is glucose dependent. GPR40 activation increases insulin secretion at high glucose levels while it does not stimulate insulin secretion at low glucose levels (56). A conversion mechanism for GPR40 activation and insulin secretion may exist. Exocytosis of insulin granules is a complex process that is regulated by membrane potential, intracellular ATP levels, intracellular signaling molecules, and [Ca^2+^]i in β-cells (57, 58). Studies showed that GPR40 activation in rat pancreatic islet β-cells results in the opening of ATP-sensitive potassium channels (K_ATP_ channels) (59, 60). It is proposed that the intracellular environment of β-cells at low glucose levels facilitates the opening of K_ATP_ channels and GPR40 activation results in the opening of K_ATP_ channels. The opening of K_ATP_ channels hyperpolarizes membrane potential and then blocks insulin secretion. When the blood glucose level is up the stimulatory concentration for insulin secretion, there may be mechanism for the blockade of GPR40-induced opening of K_ATP_ channels. It is proposed that the state of K_ATP_ channels may be the reason for the glucose-dependence of GPR40 activation to stimulate insulin secretion.

GPR40 is also expressed in islet α-cells, and GPR40 activation potentiates glucagon secretion in rodent islets *in vitro* (61, 62). GPR40 agonists elicit the oscillatory increase in [Ca^2+^]i in α-cells by activating intracellular Ca^2+^ release from ER stores and the influx of extracellular Ca^2+^, and the increase in [Ca^2+^]i triggers exocytosis of glucagon granules (63, 64). When high fat diet (HFD) without sufficient glucose is consumed, hypoglycemia may occur, provided that glucagon does not elevate while insulin secretion increases. GPR40 activation by high FFAs promotes insulin secretion to store energy substrates. Meanwhile, GPR40 activation stimulates glucagon secretion to prevent hypoglycemia. Thus, the elevation of both insulin and glucagon after GPR40 activation may be a mechanism to harmonize between the uptake of energy in the forms of FFAs and the prevention of hypoglycemia under the intake of HFD.

### GPR40 and GI hormone secretion

GPR40 is expressed in L cells, and its activation stimulates GLP-1 secretion, and the activation of Gαq/11-PLC-Ca^2+^ signaling pathway medicates GPR40-stimulated GLP-1 secretion (65). Some GPR40 agonists such as AM-1638 and AM-5262 also activate Gαs-AC-cAMP signaling pathway to potentiate GLP-1 secretion (65). GPR40 is also expressed in I cells in mouse small intestine, and GPR40 activation by LCFFAs induces the secretion of CCK in mice. The GPR40 knockout in mice leads to 50% reduction of linoleic acid-induced CCK secretion (66, 67). The Gαq/11/-PLC-Ca^2+^ signaling pathway mediates the effects of GPR40 activation on CCK secretion (66). Moreover, GPR40 is expressed in K cells and its agonists stimulate GIP secretion (68, 69).

GLP-1, CCK and GIP regulate glucose metabolism through multiple pathways. They potentiate insulin secretion by acting directly on β-cells in a glucose-dependent manner (70). Meanwhile, GLP-1 and CCK act on hypothalamus to inhibit food intake, which is a negative feedback mechanism for metabolic regulation (71). They also act on adipocytes. GLP-1 increases insulin sensitivity and promote fatty acid synthesis in adipocytes (72). Meanwhile, GLP-1 stimulates brown adipose tissue (BAT) thermogenesis and browning of white adipose tissue (WAT), which accelerate energy production and contributes to the lowering effects of GLP-1 on blood glucose levels (73–77). CCK and GIP promote fat deposit in adipocytes (78–81). The effect of CCK and GIP on fat deposit is a double-edged sword affecting glucose metabolism. To a certain extent, the induction of fat deposit may lower fatty acid levels and be beneficial to glucose metabolism. However, in the long run, it leads to obesity and insulin resistance and damage glucose metabolism. The action of GI hormones on adipocytes may be not involved in the acute regulation of blood glucose, but it may regulate glucose homeostasis in the long-term by changing the metabolic and secreting state of adipocytes.

Along with regulating adipocyte metabolism, GI hormones modulate the expression of adipokines. GLP-1 inhibits leptin expression in adipocytes. CCK antagonists increase leptin secretion from adipocytes (82). The physiological and pharmacological significance of GLP-1-inhibited leptin expression remains uncertain (83). GLP-1 and CCK upregulate adiponectin expression in adipocytes (33, 84, 85). Adiponectin exerts protective effects against inflammation and enhances insulin sensitivity in obese animals and humans (86, 87). Adiponectin also regulates glucose metabolism by stimulating fatty acids oxidation and glucose utilization in the skeletal muscle (88). The upregulation of adiponectin expression is suggested to be involved in GPR40-regulated glucose metabolism (86).

## GPR120 and glucose metabolism

**Figure 3:**
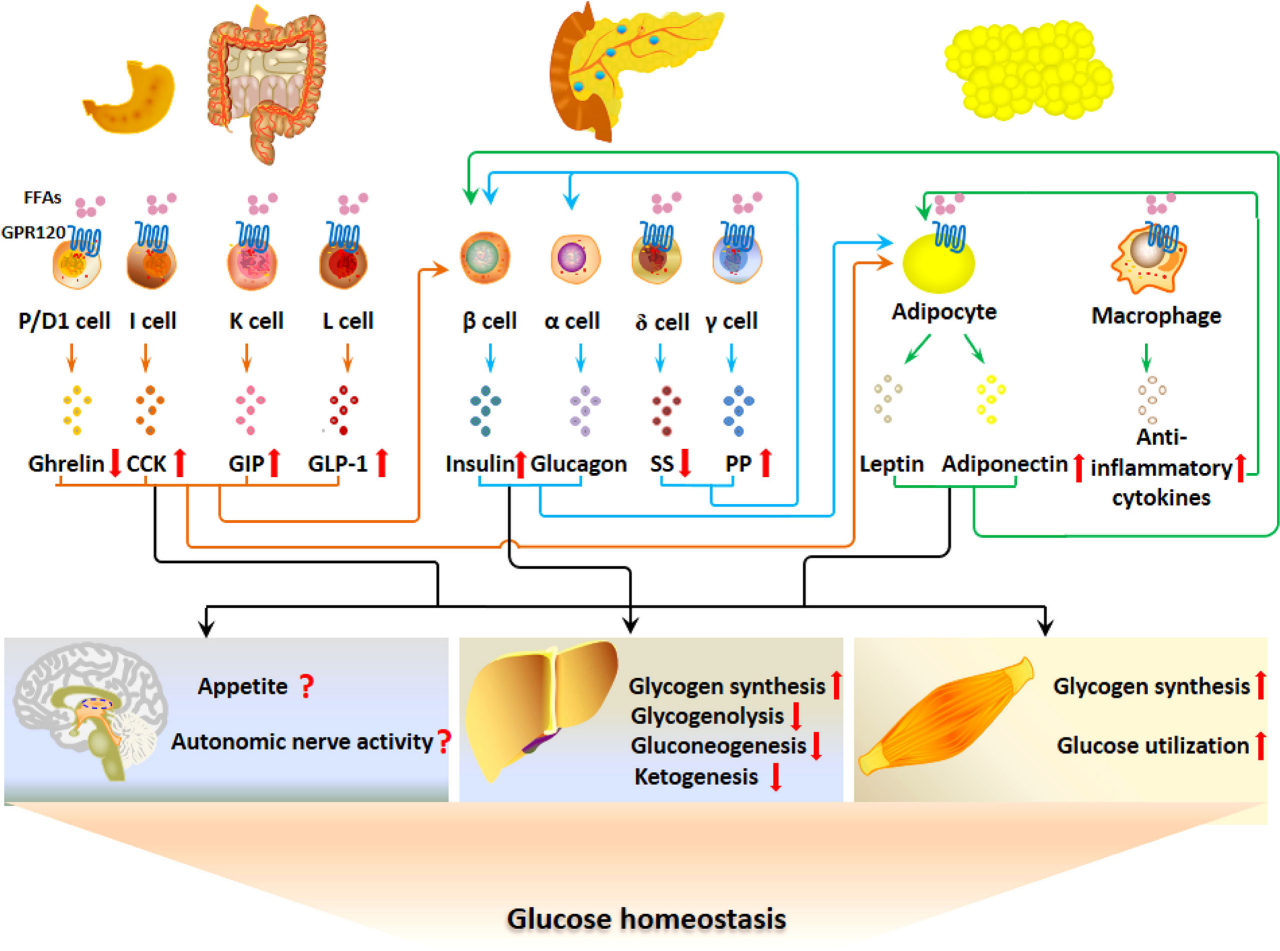
GPR120 regulates glucose metabolism *via* the G-P-A regulatory system. GPR120 activation after fat ingestion potentiates the secretion of GLP-1, CCK and GIP with the inhibition of ghrelin. Meanwhile, GPR120 activation increases adiponectin secretion. The GI hormones and adiponectin may act on CNS, liver and skeletal muscles to inhibit appetite, increase autonomous nerve activity, stimulate glycogen synthesis, and promote glucose utilization. GPR120 regulates the secretion of SS and PP in islets, which may influence the secretion of insulin in a paracrine manner. In addition, GPR120 regulates the function of adipocytes directly and indirectly *via* modulating the cytokine release from macrophages.

GPR120 is expressed in adipocytes, EECs, pancreatic islet cells, immune cells and pulmonary Clara cells (89). GPR120 was first found as an orphan receptor and later was identified as a FFA receptor in enteroendocrine L cells (7, 90). As shown in **Figure 3**, the postprandial activation of GPR120 by LCFFAs stimulates the secretion of GLP-1, CCK and GIP, with the inhibition of ghrelin secretion. The GI hormones, directly or indirectly though regulating the secretion of insulin and adipokines, regulate glucose homeostasis by acting on liver, skeletal muscle and CNS. GPR120 regulates the secretion of SS and PP in islets and then may bring about paracrine regulation of insulin and glucagon secretion. In addition, GPR120 regulates the function of adipocytes directly and indirectly *via* modulating the cytokine release from macrophages in adipose tissue. Thus, GPR120 activation excites the G-P-A regulatory system and regulates glucose metabolism.

### GPR120 and GI hormone secretion

GPR120-deficient mice developed more severe obesity and glucose intolerance when fed HFD compared with the wild type (WT) mice (91). GPR120 activation promotes GLP-1 secretion from enteroendocrine L cells *in vitro* (7). The increase in [Ca^2+^]i and the activation of extracellular signal-regulated kinase (ERK1/2) are involved in GPR120-stimulated GLP-1 secretion (7). It was suggested that GLP-1 is responsible for the acute blood glucose-lowering effects of GPR120 agonists (92). However, other reports showed that GLP-1 secretion after fat ingestion did not differ between WT mice and GPR120-knockout mice (93, 94). In GPR120 and GPR40-double knockout mice, GLP-1 secretion is not induced by oil ingestion, indicating GPR120 and GPR40 are essential for fat-induced GLP-1 secretion. These results also suggest that GPR40 activation may compensate for the defect of GPR120 in enteroendocrine L cells.

In intestine, GPR120 is also expressed in K cells and I cells, and its activation stimulates the secretion of GIP and CCK (93, 95). It has been known that FFAs stimulate CCK secretion in humans *in vivo*. Knockdown of GPR120 expression significantly attenuates FFA-induced CCK secretion (95), which indicates that GPR120 medicates the stimulatory effects of FFA on CCK secretion. Cellular analysis in STC-1 cells and GLUTag cells indicates that FFAs increase [Ca^2+^]i through the stimulation of intracellular Ca^2+^ release and extracellular Ca^2+^ influx (96). The activation of Gαq/11-PLC signaling pathway and the resultant opening of monovalent cation-specific transient receptor potential channel type 5 (TRPM5) to increase [Ca^2+^]i are responsible for GPR120-induced CCK secretion (97). GPR120 is expressed in K cells of the upper small intestine and mediate FFAs-stimulated GIP secretion in mice (93). Another study suggests that GPR120 activation stimulates CCK secretion and CCK acts on the K cells to stimulate GIP secretion (94, 98). Hormone secretion of endocrine cells such as K cells is finely controlled by many signal molecules. Some signal molecules are primary while the others are secondary, and they interrelate to regulate hormone secretion coordinately. Although the mechanism of GPR120-regulated GIP secretion is inconclusive, it is clear that GPR120 activation stimulates GIP secretion.

In stomach, GPR120 is expressed in ghrelin-secreting P/D1 cells, and its agonists inhibit ghrelin secretion in mice *in vivo* (99). GPR120 activation inhibited ghrelin secretion by activating the pertussis toxin-sensitive Gαi/o protein and inhibiting cAMP-mediated signaling pathways (100). Ghrelin is an orexigenic protein and its blood level increases during fasting to motivate ingestion. Ghrelin administration enhances appetite and increases food intake in humans and in rodents (101, 102). The inhibition of ghrelin secretion by GPR120 activation is suggested to be postprandial negative feedback to stop ingestion, which coordinates with the increase in the anorexigenic hormones such as GLP-1, CCK and GIP to regulate glucose metabolism.

Since GLP-1 and CCK inhibit appetite while ghrelin motivates ingestion, GPR120-induced changes in GI hormones are proposed to reduce appetite (103). However, GPR120 knockout mice did not show significant changes in food intake, indicating that GPR120 is physiologically dispensable for appetite regulation (91, 104). GPR120 is expressed in hypothalamus and maybe take part in unsaturated fatty acids-induced improvement of hypothalamic inflammation in obesity (105). Intracerebroventricular injection of GPR120 agonist (TUG1197) exerts anti-inflammatory activity but has no effect on body mass and caloric intake in 6-days treatment in obese mice (106). In another study, chronic intracerebroventricular injection of GPR120 agonist (GPR120 agonist III) also does not affect the intake of HFD in 15-days treatment in normal-weight mice (107). However, intracerebroventricular injection of GPR120 agonist acutely inhibits food intake in 4 hours after the administration (107). It is suggested that GPR120 plays a role in hypothalamus, but its pharmacological regulation of appetite remain to be fully investigated in the future.

### GPR120 and adipocyte function

GPR120 is expressed in white adipose tissue including subcutaneous and visceral WAT as well as BAT (108). In adipose tissue, it is expressed both in adipocytes and in macrophages (109). The expression level of GPR120 increases with the differentiation of preadipocytes. It is previously considered that GPR120 is expressed in mature adipocytes but not in preadipocytes. A study showed that GPR120 is expressed in the ciliary structure of preadipocytes and senses the extracellular FFAs and activate cAMP/EPAC (the exchange protein activated by cAMP)/CTCF (CCCTC binding factor) signaling pathway, which results in remodeling of chromosome and promotion of expression of differentiation-related genes (110). 3T3-L1 cells exhibit a low differentiation rate when GPR120 is knocked down. Although GPR120-deficient mice exhibit an obese phenotype, they have decreased differentiation of adipocytes (91, 111). It is concluded that GPR120 activation in adipose tissue promotes the differentiation of preadipocytes. The adipocyte differentiation and triglyceride accumulation may benefit the decrease in blood glucose levels *via* the promotion of glucose transformation to triglyceride. Meanwhile, GPR120 activation in macrophages inhibits the release of inflammatory factors such as interleukin-6 (IL-6), tumor necrosis factor-α (TNF-α) and interleukin-1 (IL-1) to improve insulin sensitivity of adipocytes (112, 113), which promotes glucose entry into adipocytes and inhibits FFA release to favor the control of blood glucose (114, 115). Moreover, GPR120 activation promotes the browning of WAT *via* stimulating the secretion of fibroblast growth factor 21 (FGF21), and GPR120-deficient mice have impaired browning of WAT in response to cold exposure (116). The browning of WAT increases the thermogenesis and benefits the control of postprandial blood glucose levels. GPR120 is also highly expressed in BAT with upregulation by cold exposure in mice. A study showed that GPR120-deficient neonatal mice had reduced neonatal BAT activity and thermogenesis (117). GPR120 agonists have been shown to increase fatty acid uptake and oxidation, augment mitochondrial respiration, and reduce fat mass in mice (118). Thus, the promotion of thermogenesis is one mechanism of GPR120-regulated glucose metabolism.

### GPR120 and islet hormone secretion

In pancreatic islets, GPR120 is not expressed in β-cells and α-cell. It is expressed in SS-secreting δ-cells PP-secreting γ-cells (119–122). GPR120 activation inhibits SS secretion but stimulates PP secretion in mouse islets (121, 122). The pertussis toxin-sensitive Gαi/o protein and its linked signaling pathway are suggested to mediate GPR120-inhibited SS secretion (121). The Gαq/11-PLC-Ca^2+^ signaling pathway is indicated to mediate GPR120-stimulated PP secretion (122). The physiological significance of this kind of cellular specificity of GPR120 expression in islets remains to be demonstrated. A recent study indicates the paracrine regulation of insulin secretion *via* GPR120-inhibited SS secretion (119).

## GPR43/GPR41 and glucose metabolism

### GPR43/GPR41 and hormone secretion

The SCFA receptor GPR43 and GPR41 are expressed in pancreatic β-cells and enteroendocrine L cells. GPR43-deficient mice showed a reduction of insulin secretion and developed more severe glucose intolerance when fed HFD compared with WT mice (123). GPR43 agonists increased insulin secretion *via* Gαq/11-PLC-Ca^2+^ signaling pathway in murine and human islets (123). Thus, GPR43 agonists directly act on β-cells to potentiate insulin secretion and regulate glucose metabolism. In addition, GPR43 agonists stimulate islet β-cell proliferation, and GPR43 deficiency caused a reduction in β-cell mass due to increased β-cell death (124). GPR43 agonists are suggested to enhance the compensatory capacity of β-cells to insulin resistance, which makes them potential therapeutic candidates for T2DM (123).

In contrary to GPR43, loss of GPR41 enhances glucose tolerance in mice, and GPR41 overexpression has opposite effects (125). The islets from GPR41-deficient mice have increased insulin secretion under high glucose although the islets from GPR41-overexpressing transgenic mice did not show significant changes in insulin secretion under high glucose (125). GPR41 is coupled to the Gαi/o subunit, and its activation leads to the inhibition of AC activity and the decrease in cAMP levels, which may be responsible for the reduction of insulin secretion. Therefore, although both GPR43 and GPR41 are activated by SCFAs, they mediate opposite effects on insulin secretion in β-cells. It is interesting to demonstrate the dominant type of receptors and the net effect of SCFAs on insulin secretion. GPR43/GPR41 double knockout improves glucose tolerance and insulin secretion (126). It is suggested that GPR41 has a negative but dominant effect over GPR43 and GPR43/GPR41 mediate a net inhibition on insulin secretion under normal conditions.

GPR43 is expressed in enteroendocrine L cells and mediates SCFAs-stimulated GLP-1 secretion in the mixed colonic cell cultures *in vitro* and *in vivo* (127, 128). GPR43-deficient mice show reduced SCFAs-induced GLP-1 secretion and impaired glucose tolerance (127). Gαq/11-PLC-Ca^2+^ signaling pathway was reported to mediate the effects of GPR43 activation on GLP-1 secretion in L cells (127). Although GPR41 is expressed in enteroendocrine L cells (129), its role in GLP-1 secretion remains to be demonstrated.

### GPR43/GPR41 and adipocyte function

GPR43 is expressed in adipocytes, but its role in adipocytes is not clear (130). GPR43 expression levels in WAT are higher in HFD-induced obese mice than in normal chow-fed mice. It was reported that SCFAs treatment suppresses lipolysis in 3T3-L1 adipocytes and adipocytes isolated from mice adipose tissue and that GPR43 knockdown inhibits adipogenesis (131). However, another report showed that GPR43-deficient mice tend to become obese easier when fed HFD than WT mice and GPR43 overexpression in adipose tissue leads to the lean phenotype in mice (132). Further studies are needed to elucidate the role of GPR43 in white adipose tissue. As to BAT, the other type of adipose tissue, GPR43-deficient old age mice exhibit the increase in BAT activity and increased energy expenditure, which may be responsible for the improved insulin sensitivity in the mice (133). GPR43 mediates the stimulatory effects of SCFAs on adipogenesis and mitochondrial biogenesis in brown adipocytes (134). It is proposed that the stimulation of BAT contributes to the lean phenotype of GPR43 overexpression. However, GPR43 expression in adipose tissue is not different between obese patients and lean subjects, and GPR43 agonists do not induce the differentiation of human preadipocytes isolated from omental adipose tissue (135). This study indicates that a species difference has to be considered between humans and mice in the study of GPR43 actions.

The role of GPR41 in regulating adipocytes function is also unsettled. GPR41 expression was found in adipocytes, and its activation stimulated leptin secretion from adipose tissues (136). However, other studies did not detect GPR41 expression in mouse adipose tissue, and the stimulation of leptin secretion by SCFA is suggested to be mediated by GPR43 rather than GPR41 (137). Male GPR41-deficient mice show higher body fat mass and plasma leptin levels as well as higher glucose levels than WT mice (138). Although this study does not resolve the controversial about the expression of GPR41 in adipocytes, it demonstrates that GPR41 surely regulates fat and glucose metabolism *via* direct or indirect actions on adipocytes. Immune cells such as macrophages distribute in adipose tissue and modulate adipocyte function *via* paracrine signaling cytokines such as IL-6, IL-1, and TNF-α (139, 140). GPR41 and GPR43 are expressed in macrophages (141, 142). Therefore, the involvement of macrophages in the regulation of fat accumulation and adipokine secretion in adipocytes may complicate the observation of GPR43/GPR41-regulated adipocyte function.

### Differences between SCFAs AND LCFFAs in metabolic regulation

SCFAs are very different to LCFFAs in characteristics and its source in human body. SCFAs in humans are mainly obtained from colon as the products of bacterial fermentation from insoluble fiber and proteins but not from food intake (143, 144). Thus, the physiological significance of SCFAs in regulating metabolism is surely different to LCFFAs. Although both SCFA receptors and LCFFA receptor are involved in the regulation of glucose metabolism through targeting the secretion of insulin and GLP-1 as well as the function of adipocytes, they should have distinct effects on glucose metabolism in physiological and pathophysiological conditions. The details in differences of FFA receptors in glucose metabolism in different metabolic states are worth of further exploration.

## Targeting FFA receptors for drug development

The development of drugs targeting FFA receptors has been going on for decades, and the earliest is the development of GPR40 agonists for the treatment of T2DM. GPR40 agonist TAK-875 exhibits the ability to improve blood glucose control in patients with T2DM. However, it stopped in clinical trial III because of its hepatotoxicity (145). The other GPR40 agonists including LY2881835, AMG837, CPL207280, SCO-267, CPU-014 and AM-1638 are in the pipeline of drug development for T2DM treatment. Eli Lilly and Amgen initiated phase I/II clinical trials with LY2881835 and AMG837, respectively (146). Interestingly, GPR40 antagonists also have been developed for T2DM treatment. GPR40 antagonist DC260126 inhibits LCFA-stimulated increased in [Ca^2+^]i and protect β-cells against palmitate-induced ER stress and cell apoptosis (147, 148).

GPR120 agonists have been shown to improve insulin sensitivity in obese subjects. GPR120 agonist TUG-891 has been indicated as therapeutic agent of diabetes and obesity (8, 146). The other GPR120 agonists including NCG75, GSK137647A, AZ13581837 and CpdA all improve glucose tolerance in HFD-induced obese mice by increasing insulin sensitivity (146, 149, 150). Although a number of GPR120 agonists have been discovered, they have not moved to clinical trials. The insufficiency in both the understanding of GPR120 biology and the discovery of specific agonists with efficient effects *in vivo* may obstruct the development of drugs targeting GPR120.

GPR43 not only regulates glucose metabolism but also plays an important role in regulating immune function. GPR43 antagonists have been in development for anti-inflammation (151, 152). Since patients of T2DM are in the state of noninfectious microinflammation in multiple tissues such as adipose tissue, heart and liver (153), it is proposed that GPR43 antagonists may improve T2DM through anti-inflammation. GPR41 is also the therapeutic target for inflammatory diseases, but the development of drugs targeting GPR41 is relatively few compared with the other FFA receptors. There is still a long way to go for the development of drugs targeting GPR43/GPR41 for the treatment of metabolic diseases such as T2DM.

## Conclusion and prospect

FFA receptors distribute in metabolism-related tissues to sense the fluctuation in extracellular FFAs and then regulate glucose metabolism through G-P-A regulatory system. The overlapping distribution of different types of FFA receptors in intestine indicates the importance of FFA receptors in nutrient sensing and metabolic regulation. This phenomenon also suggests that different FFA receptors may function differently and are distinguishingly activated in different nutritional states such as food intake, fasting, and obesity. Thus, the cells are able to response specifically to the changes in the level and composition of blood FFAs, which ensures the optimal fine-tuning of regulatory system for the maintenance of metabolism homeostasis. To date, the research in FFA receptor activation by different FFAs *in vivo* under different nutritional states is deficient. In the future, the detailed analysis of FFA receptor activation in different nutritional states will increase the understanding of FFA-regulated metabolism.

The regulatory effects of ligand-receptor interaction depend on not only the levels of ligands but also the levels of receptors. The changes in cellular expression of FFA receptors certainly influence the actions of FFAs on metabolism. It was found that HFD-induced obesity leads to the downregulation of GPR120 in intestine and in pancreatic islets in mice (122, 154). Due to the differentiation of adipocytes, GPR120 levels in subcutaneous fat and omental fat are increased in obese human subjects compared with those in lean subjects (91). However, morbidly obese human subjects (BMI 54.0 ± 5.7 kg/m^2^) have lower GPR120 levels in visceral adipose tissue than nonobese subjects, indicating that the enlarged adipocytes goes to the other side for GPR120 expression (154). Demonstration of the expression level of FFA receptors in different nutritional states is essential for understanding their physiological and pathophysiological role and the strategies to regulate metabolism through FFA receptors. It is suggested that compounds that upregulate the expression of FFA receptors may improve metabolic disorders synergistically with FFA receptor agonists. A recent study shows that PPAR-γ agonist upregulates GPR120 expression in adipocytes and synergistically enhances the effects of GPR120 agonists on metabolism (114). This study gives an example of how-to strength FFA receptors-FFAs interaction to regulate metabolism. More studies are expected to demonstrate the regulatory mechanism of the expression of FFA receptors in the future.

## Author contributions

The author confirms being the sole contributor of this work and has approved it for publication.

## Funding

This work was supported by the Funding from Innovative Group of Xi’an Medical University (No. 2021TD01).

## Conflict of interest

The authors declares that the research was conducted in the absence of any commercial or financial relationships that could be construed as a potential conflict of interest.

## Publisher’s note

All claims expressed in this article are solely those of the authors and do not necessarily represent those of their affiliated organizations, or those of the publisher, the editors and the reviewers. Any product that may be evaluated in this article, or claim that may be made by its manufacturer, is not guaranteed or endorsed by the publisher.

## References

[1] Wachsmuth HR Weninger SN Duca FA. Role of the gut-brain axis in energy and glucose metabolism. Exp Mol Med (2022) 54:377–92. doi: 10.1038/s12276-021-00677-w PMC9076644 35474341

[2] Holst JJ Gribble F Horowitz M Rayner CK. Roles of the gut in glucose homeostasis. Diabetes Care (2016) 39:884–92. doi: 10.2337/dc16-0351 27222546

[3] Kim W Egan JM. The role of incretins in glucose homeostasis and diabetes treatment. Pharmacol Rev (2008) 60:470–512. doi: 10.1124/pr.108.000604 19074620 PMC2696340

[4] Walker JT Saunders DC Brissova M Powers AC. The human islet: Mini-organ with mega-impact. Endocrine Rev (2021) 42:605–57. doi: 10.1210/endrev/bnab010 PMC8476939 33844836

[5] Röder PV Wu B Liu Y Han W. Pancreatic regulation of glucose homeostasis. Exp Mol Med (2016) 48:e219. doi: 10.1038/emm.2016.6 26964835 PMC4892884

[6] Chueire VB Muscelli E. Effect of free fatty acids on insulin secretion, insulin sensitivity and incretin effect - a narrative review. Arch Endocrinol Metab (2021) 65:24–31. doi: 10.20945/2359-3997000000313 33320449 PMC10528699

[7] Hirasawa A Tsumaya K Awaji T Katsuma S Adachi T Yamada M. Free fatty acids regulate gut incretin glucagon-like peptide-1 secretion through GPR120. Nat Med (2005) 11:90–4. doi: 10.1038/nm1168 15619630

[8] Kimura I Ichimura A Ohue-Kitano R Igarashi M. Free fatty acid receptors in health and disease. Physiol Rev (2020) 100:171–210. doi: 10.1152/physrev.00041.2018 31487233

[9] Miyamoto J Hasegawa S Kasubuchi M Ichimura A Nakajima A Kimura I. Nutritional signaling *via* free fatty acid receptors. Int J Mol Sci (2016) 17:450. doi: 10.3390/ijms17040450 27023530 PMC4848906

[10] Kebede MA Alquier T Latour MG Poitout V. Lipid receptors and islet function: therapeutic implications? Diabetes Obes Metab (2009) 11 Suppl 4:10–20. doi: 10.1111/j.1463-1326.2009.01114.x PMC2889915 19817784

[11] Norton L Shannon C Gastaldelli A DeFronzo RA. Insulin: The master regulator of glucose metabolism. Metabolism: Clin Exp (2022) 129:155142. doi: 10.1016/j.metabol.2022.155142 35066003

[12] Gilon P. The role of α-cells in islet function and glucose homeostasis in health and type 2 diabetes. J Mol Biol (2020) 432:1367–94. doi: 10.1016/j.jmb.2020.01.004 31954131

[13] Huising MO. Paracrine regulation of insulin secretion. Diabetologia (2020) 63:2057–63. doi: 10.1007/s00125-020-05213-5 PMC7968070 32894316

[14] Holst JJ Gasbjerg LS Rosenkilde MM. The role of incretins on insulin function and glucose homeostasis. Endocrinology (2021) 162:bqab065. doi: 10.1210/endocr/bqab065 33782700 PMC8168943

[15] Gerst F Wagner R Oquendo MB Siegel-Axel D Fritsche A Heni M. What role do fat cells play in pancreatic tissue? Mol Metab (2019) 25:1–10. doi: 10.1016/j.molmet.2019.05.001 31113756 PMC6600604

[16] Osinski C Moret D Clément K Serradas P Ribeiro A. Enteroendocrine system and gut barrier in metabolic disorders. Int J Mol Sci (2022) 23:3732. doi: 10.3390/ijms23073732 35409092 PMC8998765

[17] Rosendo-Silva D Matafome P. Gut-adipose tissue crosstalk: A bridge to novel therapeutic targets in metabolic syndrome? Obes Rev an Off J Int Assoc Study Obes (2021) 22:e13130. doi: 10.1111/obr.13130 32815267

[18] Guccio N Gribble FM Reimann F. Glucose-dependent insulinotropic polypeptide-a postprandial hormone with unharnessed metabolic potential. Annu Rev Nutr (2022) 42:21–44. doi: 10.1146/annurev-nutr-062320-113625 35609956

[19] Kamohara S Burcelin R Halaas JL Friedman JM Charron MJ. Acute stimulation of glucose metabolism in mice by leptin treatment. Nature (1997) 389:374–7. doi: 10.1038/38717 9311777

[20] Zhao YF Feng DD Chen C. Contribution of adipocyte-derived factors to beta-cell dysfunction in diabetes. Int J Biochem Cell Biol (2006) 38:804–19. doi: 10.1016/j.biocel.2005.11.008 16378747

[21] Pereira S Cline DL Glavas MM Covey SD Kieffer TJ. Tissue-specific effects of leptin on glucose and lipid metabolism. Endocrine Rev (2021) 42:1–28. doi: 10.1210/endrev/bnaa027 33150398 PMC7846142

[22] Schwartz MW Baskin DG Bukowski TR Kuijper JL Foster D Lasser G. Specificity of leptin action on elevated blood glucose levels and hypothalamic neuropeptide y gene expression in ob/ob mice. Diabetes (1996) 45:531–5. doi: 10.2337/diab.45.4.531 8603777

[23] Coppari R Bjørbæk C. Leptin revisited: its mechanism of action and potential for treating diabetes. Nat Rev Drug Discovery (2012) 11:692–708. doi: 10.1038/nrd3757 22935803 PMC4019022

[24] Berglund ED Vianna CR Donato J Jr. Kim MH Chuang JC Lee CE. Direct leptin action on POMC neurons regulates glucose homeostasis and hepatic insulin sensitivity in mice. J Clin Invest (2012) 122:1000–9. doi: 10.1172/jci59816 PMC3287225 22326958

[25] Cummings BP Bettaieb A Graham JL Stanhope KL Dill R Morton GJ. Subcutaneous administration of leptin normalizes fasting plasma glucose in obese type 2 diabetic UCD-T2DM rats. Proc Natl Acad Sci United States America (2011) 108:14670–5. doi: 10.1073/pnas.1107163108 PMC3167517 21873226

[26] Mendoza-Herrera K Florio AA Moore M Marrero A Tamez M Bhupathiraju SN. The leptin system and diet: A mini review of the current evidence. Front Endocrinol (2021) 12:749050. doi: 10.3389/fendo.2021.749050 PMC8651558 34899599

[27] Wang C Du X Fu F Li X Wang Z Zhou Y. Adiponectin gene therapy prevents islet loss after transplantation. J Cell Mol Med (2022) 26:4847–58. doi: 10.1111/jcmm.17515 PMC9465193 35975481

[28] Rakatzi I Mueller H Ritzeler O Tennagels N Eckel J. Adiponectin counteracts cytokine- and fatty acid-induced apoptosis in the pancreatic beta-cell line INS-1. Diabetologia (2004) 47:249–58. doi: 10.1007/s00125-003-1293-3 14722646

[29] Holland WL Miller RA Wang ZV Sun K Barth BM Bui HH. Receptor-mediated activation of ceramidase activity initiates the pleiotropic actions of adiponectin. Nat Med (2011) 17:55–63. doi: 10.1038/nm.2277 21186369 PMC3134999

[30] Yanai H Yoshida H. Beneficial effects of adiponectin on glucose and lipid metabolism and atherosclerotic progression: Mechanisms and perspectives. Int J Mol Sci (2019) 20:1190. doi: 10.3390/ijms20051190 PMC6429491 30857216

[31] Marques-Oliveira GH Silva TM Lima WG Valadares HMS Chaves VE. Insulin as a hormone regulator of the synthesis and release of leptin by white adipose tissue. Peptides (2018) 106:49–58. doi: 10.1016/j.peptides.2018.06.007 29953915

[32] Li L Miao Z Liu R Yang M Liu H Yang G. Liraglutide prevents hypoadiponectinemia-induced insulin resistance and alterations of gene expression involved in glucose and lipid metabolism. Mol Med (Cambridge Mass.) (2011) 17:1168–78. doi: 10.2119/molmed.2011.00051 PMC3321815 21785811

[33] Wang A Li T An P Yan W Zheng H Wang B. Exendin-4 upregulates adiponectin level in adipocytes *via* Sirt1/Foxo-1 signaling pathway. PloS One (2017) 12:e0169469. doi: 10.1371/journal.pone.0169469 28122026 PMC5266308

[34] Naitoh R Miyawaki K Harada N Mizunoya W Toyoda K Fushiki T. Inhibition of GIP signaling modulates adiponectin levels under high-fat diet in mice. Biochem Biophys Res Commun (2008) 376:21–5. doi: 10.1016/j.bbrc.2008.08.052 18723001

[35] Huda MS Wilding JP Pinkney JH. Gut peptides and the regulation of appetite. Obes Rev an Off J Int Assoc Study Obes (2006) 7:163–82. doi: 10.1111/j.1467-789X.2006.00245.x 16629873

[36] Warnotte C Gilon P Nenquin M Henquin JC. Mechanisms of the stimulation of insulin release by saturated fatty acids. A study palmitate effects Mouse beta-cells. Diabetes (1994) 43:703–11. doi: 10.2337/diab.43.5.703 8168648

[37] Haber EP Procópio J Carvalho CR Carpinelli AR Newsholme P Curi R. New insights into fatty acid modulation of pancreatic beta-cell function. Int Rev cytology (2006) 248:1–41. doi: 10.1016/s0074-7696(06)48001-3 16487789

[38] Dimitriadis GD Maratou E Kountouri A Board M Lambadiari V. Regulation of postabsorptive and postprandial glucose metabolism by insulin-dependent and insulin-independent mechanisms: An integrative approach. Nutrients (2021) 13:159. doi: 10.3390/nu13010159 PMC7825450 33419065

[39] Mason TM Goh T Tchipashvili V Sandhu H Gupta N Lewis GF. Prolonged elevation of plasma free fatty acids desensitizes the insulin secretory response to glucose *in vivo* in rats. Diabetes (1999) 48:524–30. doi: 10.2337/diabetes.48.3.524 10078552

[40] Unger RH. Lipotoxicity in the pathogenesis of obesity-dependent NIDDM. Genet Clin implications. Diabetes (1995) 44:863–70. doi: 10.2337/diab.44.8.863 7621989

[41] Shimabukuro M Zhou YT Levi M Unger RH. Fatty acid-induced beta cell apoptosis: a link between obesity and diabetes. Proc Natl Acad Sci United States America (1998) 95:2498–502. doi: 10.1073/pnas.95.5.2498 PMC19389 9482914

[42] Dobbins RL Chester MW Stevenson BE Daniels MB Stein DT McGarry JD. A fatty acid- dependent step is critically important for both glucose- and non-glucose-stimulated insulin secretion. J Clin Invest (1998) 101:2370–6. doi: 10.1172/jci1813 PMC508826 9616208

[43] Dobbins RL Chester MW Daniels MB McGarry JD Stein DT. Circulating fatty acids are essential for efficient glucose-stimulated insulin secretion after prolonged fasting in humans. Diabetes (1998) 47:1613–8. doi: 10.2337/diabetes.47.10.1613 9753300

[44] Gilman AG. G Proteins: transducers of receptor-generated signals. Annu Rev Biochem (1987) 56:615–49. doi: 10.1146/annurev.bi.56.070187.003151 3113327

[45] Simonds WF. G Protein regulation of adenylate cyclase. Trends Pharmacol Sci (1999) 20:66–73. doi: 10.1016/s0165-6147(99)01307-3 10101967

[46] Rhee SG. Regulation of phosphoinositide-specific phospholipase c. Annu Rev Biochem (2001) 70:281–312. doi: 10.1146/annurev.biochem.70.1.281 11395409 PMC4781088

[47] Aydin Y Coin I. Biochemical insights into structure and function of arrestins. FEBS J (2021) 288:2529–49. doi: 10.1111/febs.15811 33690974

[48] Kim K Han Y Duan L Chung KY. Scaffolding of mitogen-activated protein kinase signaling by β-arrestins. Int J Mol Sci (2022) 23:1000. doi: 10.3390/ijms23021000 35055186 PMC8778048

[49] Itoh Y Kawamata Y Harada M Kobayashi M Fujii R Fukusumi S. Free fatty acids regulate insulin secretion from pancreatic beta cells through GPR40. Nature (2003) 422:173–6. doi: 10.1038/nature01478 12629551

[50] Kebede M Alquier T Latour MG Semache M Tremblay C Poitout V. The fatty acid receptor GPR40 plays a role in insulin secretion *in vivo* after high-fat feeding. Diabetes (2008) 57:2432–7. doi: 10.2337/db08-0553 PMC2518494 18559658

[51] Fujiwara K Maekawa F Yada T. Oleic acid interacts with GPR40 to induce Ca2+ signaling in rat islet beta-cells: mediation by PLC and l-type Ca2+ channel and link to insulin release. Am J Physiol Endocrinol Metab (2005) 289:E670–677. doi: 10.1152/ajpendo.00035.2005 15914509

[52] Shapiro H Shachar S Sekler I Hershfinkel M Walker MD. Role of GPR40 in fatty acid action on the beta cell line INS-1E. Biochem Biophys Res Commun (2005) 335:97–104. doi: 10.1016/j.bbrc.2005.07.042 16081037

[53] Schnell S Schaefer M Schöfl C. Free fatty acids increase cytosolic free calcium and stimulate insulin secretion from beta-cells through activation of GPR40. Mol Cell Endocrinol (2007) 263:173–80. doi: 10.1016/j.mce.2006.09.013 17101212

[54] Yamada H Yoshida M Ito K Dezaki K Yada T Ishikawa SE. Potentiation of glucose-stimulated insulin secretion by the GPR40-PLC-TRPC pathway in pancreatic β-cells. Sci Rep (2016) 6:25912. doi: 10.1038/srep25912 27180622 PMC4867641

[55] Feng DD Luo Z Roh SG Hernandez M Tawadros N Keating DJ. Reduction in voltage-gated k+ currents in primary cultured rat pancreatic beta-cells by linoleic acids. Endocrinology (2006) 147:674–82. doi: 10.1210/en.2005-0225 16254037

[56] Sakuma K Yabuki C Maruyama M Abiru A Komatsu H Negoro N. Fasiglifam (TAK-875) has dual potentiating mechanisms *via* gαq-GPR40/FFAR1 signaling branches on glucose-dependent insulin secretion. Pharmacol Res Perspect (2016) 4:e00237. doi: 10.1002/prp2.237 27433346 PMC4876146

[57] Vakilian M Tahamtani Y Ghaedi K. A review on insulin trafficking and exocytosis. Gene (2019) 706:52–61. doi: 10.1016/j.gene.2019.04.063 31039435

[58] Röder PV Wong X Hong W Han W. Molecular regulation of insulin granule biogenesis and exocytosis. Biochem J (2016) 473:2737–56. doi: 10.1042/bcj20160291 27621482

[59] Zhao YF Wang L Zha D Qiao L Lu L Yu J. GW9508 inhibits insulin secretion by activating ATP-sensitive potassium channels in rat pancreatic β-cells. J Mol Endocrinol (2013) 51:69–77. doi: 10.1530/jme-13-0019 23628491

[60] Zhao YF Pei J Chen C. Activation of ATP-sensitive potassium channels in rat pancreatic beta-cells by linoleic acid through both intracellular metabolites and membrane receptor signalling pathway. J Endocrinol (2008) 198:533–40. doi: 10.1677/joe-08-0105 18550787

[61] Ueno H Ito R Abe SI Ookawara M Miyashita H Ogino H. SCO-267, a GPR40 full agonist, improves glycemic and body weight control in rat models of diabetes and obesity. J Pharmacol Exp Ther (2019) 370:172–81. doi: 10.1124/jpet.118.255885 31182471

[62] Kristinsson H Sargsyan E Manell H Smith DM Göpel SO Bergsten P. Basal hypersecretion of glucagon and insulin from palmitate-exposed human islets depends on FFAR1 but not decreased somatostatin secretion. Sci Rep (2017) 7:4657. doi: 10.1038/s41598-017-04730-5 28680093 PMC5498543

[63] Flodgren E Olde B Meidute-Abaraviciene S Winzell MS Ahrén B Salehi A. GPR40 is expressed in glucagon producing cells and affects glucagon secretion. Biochem Biophys Res Commun (2007) 354:240–5. doi: 10.1016/j.bbrc.2006.12.193 17214971

[64] Wang L Zhao Y Gui B Fu R Ma F Yu J. Acute stimulation of glucagon secretion by linoleic acid results from GPR40 activation and [Ca2+]i increase in pancreatic islet {alpha}-cells. J Endocrinol (2011) 210:173–9. doi: 10.1530/joe-11-0132 21565851

[65] Hauge M Vestmar MA Husted AS Ekberg JP Wright MJ Di Salvo J. GPR40 (FFAR1) - combined gs and gq signaling *in vitro* is associated with robust incretin secretagogue action *ex vivo* and *in vivo* . Mol Metab (2015) 4:3–14. doi: 10.1016/j.molmet.2014.10.002 25685685 PMC4314522

[66] Liou AP Lu X Sei Y Zhao X Pechhold S Carrero RJ. The G-protein-coupled receptor GPR40 directly mediates long-chain fatty acid-induced secretion of cholecystokinin. Gastroenterology (2011) 140:903–12. doi: 10.1053/j.gastro.2010.10.012 PMC4717904 20955703

[67] Kato T Harada N Ikeguchi-Ogura E Sankoda A Hatoko T Lu X. Gene expression of nutrient-sensing molecules in I cells of CCK reporter male mice. J Mol Endocrinol (2021) 66:11–22. doi: 10.1530/jme-20-0134 33151898

[68] Sankoda A Harada N Kato T Ikeguchi E Iwasaki K Yamane S. Free fatty acid receptors, G protein-coupled receptor 120 and G protein-coupled receptor 40, are essential for oil-induced gastric inhibitory polypeptide secretion. J Diabetes Invest (2019) 10:1430–7. doi: 10.1111/jdi.13059 PMC6825923 31002464

[69] Edfalk S Steneberg P Edlund H. Gpr40 is expressed in enteroendocrine cells and mediates free fatty acid stimulation of incretin secretion. Diabetes (2008) 57:2280–7. doi: 10.2337/db08-0307 PMC2518478 18519800

[70] Rossetti L Shulman GI Zawalich WS. Physiological role of cholecystokinin in meal-induced insulin secretion in conscious rats. studies with l 364718, a specific inhibitor of CCK-receptor binding. Diabetes (1987) 36:1212–5. doi: 10.2337/diab.36.10.1212 3308589

[71] Yu Y Fernandez ID Meng Y Zhao W Groth SW. Gut hormones, adipokines, and pro- and anti-inflammatory cytokines/markers in loss of control eating: A scoping review. Appetite (2021) 166:105442. doi: 10.1016/j.appet.2021.105442 34111480 PMC10683926

[72] Egan JM Montrose-Rafizadeh C Wang Y Bernier M Roth J. Glucagon-like peptide-1(7-36) amide (GLP-1) enhances insulin-stimulated glucose metabolism in 3T3-L1 adipocytes: one of several potential extrapancreatic sites of GLP-1 action. Endocrinology (1994) 135:2070–5. doi: 10.1210/endo.135.5.7956929 7956929

[73] Beiroa D Imbernon M Gallego R Senra A Herranz D Villarroya F. GLP-1 agonism stimulates brown adipose tissue thermogenesis and browning through hypothalamic AMPK. Diabetes (2014) 63:3346–58. doi: 10.2337/db14-0302 24917578

[74] López M Diéguez C Nogueiras R. Hypothalamic GLP-1: the control of BAT thermogenesis and browning of white fat. Adipocyte (2015) 4:141–5. doi: 10.4161/21623945.2014.983752 PMC4497297 26167417

[75] Xu F Lin B Zheng X Chen Z Cao H Xu H. GLP-1 receptor agonist promotes brown remodelling in mouse white adipose tissue through SIRT1. Diabetologia (2016) 59:1059–69. doi: 10.1007/s00125-016-3896-5 26924394

[76] Li H Donelan W Wang F Zhang P Yang L Ding Y. GLP-1 induces the expression of FNDC5 derivatives that execute lipolytic actions. Front Cell Dev Biol (2021) 9:777026. doi: 10.3389/fcell.2021.777026 34869379 PMC8636013

[77] Zhou JY Poudel A Welchko R Mekala N Chandramani-Shivalingappa P Rosca MG. Liraglutide improves insulin sensitivity in high fat diet induced diabetic mice through multiple pathways. Eur J Pharmacol (2019) 861:172594. doi: 10.1016/j.ejphar.2019.172594 31412267

[78] Plaza A Merino B Cano V Domínguez G Pérez-Castells J Fernández-Alfonso MS. Cholecystokinin is involved in triglyceride fatty acid uptake by rat adipose tissue. J Endocrinol (2018) 236:137–50. doi: 10.1530/joe-17-0580 29339381

[79] Lo CM King A Samuelson LC Kindel TL Rider T Jandacek RJ. Cholecystokinin knockout mice are resistant to high-fat diet-induced obesity. Gastroenterology (2010) 138:1997–2005. doi: 10.1053/j.gastro.2010.01.044 20117110 PMC3049264

[80] Yip RG Boylan MO Kieffer TJ Wolfe MM. Functional GIP receptors are present on adipocytes. Endocrinology (1998) 139:4004–7. doi: 10.1210/endo.139.9.6288 9724057

[81] Weaver RE Donnelly D Wabitsch M Grant PJ Balmforth AJ. Functional expression of glucose-dependent insulinotropic polypeptide receptors is coupled to differentiation in a human adipocyte model. Int J Obes (2005) 2008) 32:1705–11. doi: 10.1038/ijo.2008.148 18779825

[82] Attoub S Levasseur S Buyse M Goïot H Laigneau JP Moizo L. Physiological role of cholecystokinin b/gastrin receptor in leptin secretion. Endocrinology (1999) 140:4406–10. doi: 10.1210/endo.140.10.7079 10499492

[83] Zhou B Dong C Zhao B Su X Luo Y Xie L. (E(X)-4)(2)-Fc, an effective long-acting GLP-1 receptor agonist, reduces obesity-related inflammation by inhibiting leptin expression. Biochem Biophys Res Commun (2020) 529:562–8. doi: 10.1016/j.bbrc.2020.06.054 32736674

[84] Singh A Fernandes JRD Chhabra G Krishna A Banerjee A. Liraglutide modulates adipokine expression during adipogenesis, ameliorating obesity, and polycystic ovary syndrome in mice. Endocrine (2019) 64:349–66. doi: 10.1007/s12020-019-01891-3 30904998

[85] Plaza A Merino B Del Olmo N Ruiz-Gayo M. The cholecystokinin receptor agonist, CCK-8, induces adiponectin production in rat white adipose tissue. Br J Pharmacol (2019) 176:2678–90. doi: 10.1111/bph.14690 PMC6609540 31012948

[86] Khoramipour K Chamari K Hekmatikar AA Ziyaiyan A Taherkhani S Elguindy NM. Adiponectin: Structure, physiological functions, role in diseases, and effects of nutrition. Nutrients (2021) 13:1180. doi: 10.3390/nu13041180 33918360 PMC8066826

[87] Awazawa M Ueki K Inabe K Yamauchi T Kubota N Kaneko K. Adiponectin enhances insulin sensitivity by increasing hepatic IRS-2 expression *via* a macrophage-derived IL-6-dependent pathway. Cell Metab (2011) 13:401–12. doi: 10.1016/j.cmet.2011.02.010 21459325

[88] Abou-Samra M Selvais CM Dubuisson N Brichard SM. Adiponectin and its mimics on skeletal muscle: Insulin sensitizers, fat burners, exercise mimickers, muscling pills … or everything together? Int J Mol Sci (2020) 21:2620. doi: 10.3390/ijms21072620 PMC7178193 32283840

[89] Miyauchi S Hirasawa A Iga T Liu N Itsubo C Sadakane K. Distribution and regulation of protein expression of the free fatty acid receptor GPR120. Naunyn-Schmiedeberg's Arch Pharmacol (2009) 379:427–34. doi: 10.1007/s00210-008-0390-8 19145429

[90] Fredriksson R Höglund PJ Gloriam DE Lagerström MC Schiöth HB. Seven evolutionarily conserved human rhodopsin G protein-coupled receptors lacking close relatives. FEBS Lett (2003) 554:381–8. doi: 10.1016/s0014-5793(03)01196-7 14623098

[91] Ichimura A Hirasawa A Poulain-Godefroy O Bonnefond A Hara T Yengo L. Dysfunction of lipid sensor GPR120 leads to obesity in both mouse and human. Nature (2012) 483:350–4. doi: 10.1038/nature10798 22343897

[92] Sundström L Myhre S Sundqvist M Ahnmark A McCoull W Raubo P. The acute glucose lowering effect of specific GPR120 activation in mice is mainly driven by glucagon-like peptide 1. PloS One (2017) 12:e0189060. doi: 10.1371/journal.pone.0189060 29206860 PMC5716539

[93] Iwasaki K Harada N Sasaki K Yamane S Iida K Suzuki K. Free fatty acid receptor GPR120 is highly expressed in enteroendocrine K cells of the upper small intestine and has a critical role in GIP secretion after fat ingestion. Endocrinology (2015) 156:837–46. doi: 10.1210/en.2014-1653 25535828

[94] Sankoda A Harada N Iwasaki K Yamane S Murata Y Shibue K. Long-chain free fatty acid receptor GPR120 mediates oil-induced GIP secretion through CCK in Male mice. Endocrinology (2017) 158:1172–80. doi: 10.1210/en.2017-00090 28324023

[95] Tanaka T Katsuma S Adachi T Koshimizu TA Hirasawa A Tsujimoto G. Free fatty acids induce cholecystokinin secretion through GPR120. Naunyn-Schmiedeberg's Arch Pharmacol (2008) 377:523–7. doi: 10.1007/s00210-007-0200-8 17972064

[96] Sidhu SS Thompson DG Warhurst G Case RM Benson RS. Fatty acid-induced cholecystokinin secretion and changes in intracellular Ca2+ in two enteroendocrine cell lines, STC-1 and GLUTag. J Physiol (2000) 528 Pt 1:165–76. doi: 10.1111/j.1469-7793.2000.00165.x PMC2270123 11018115

[97] Shah BP Liu P Yu T Hansen DR Gilbertson TA. TRPM5 is critical for linoleic acid-induced CCK secretion from the enteroendocrine cell line, STC-1. American journal of physiology. Cell Physiol (2012) 302:C210–219. doi: 10.1152/ajpcell.00209.2011 PMC3328913 21998136

[98] Murata Y Harada N Kishino S Iwasaki K Ikeguchi-Ogura E Yamane S. Medium-chain triglycerides inhibit long-chain triglyceride-induced GIP secretion through GPR120-dependent inhibition of CCK. iScience (2021) 24:102963. doi: 10.1016/j.isci.2021.102963 34466786 PMC8382997

[99] Lu X Zhao X Feng J Liou AP Anthony S Pechhold S. Postprandial inhibition of gastric ghrelin secretion by long-chain fatty acid through GPR120 in isolated gastric ghrelin cells and mice. American journal of physiology. Gastroint liver Physiol (2012) 303:G367–376. doi: 10.1152/ajpgi.00541.2011 PMC3774249 22678998

[100] Engelstoft MS Park WM Sakata I Kristensen LV Husted AS Osborne-Lawrence S. Seven transmembrane G protein-coupled receptor repertoire of gastric ghrelin cells. Mol Metab (2013) 2:376–92. doi: 10.1016/j.molmet.2013.08.006 PMC3854997 24327954

[101] Tschöp M Smiley DL Heiman ML. Ghrelin induces adiposity in rodents. Nature (2000) 407:908–13. doi: 10.1038/35038090 11057670

[102] Wren AM Seal LJ Cohen MA Brynes AE Frost GS Murphy KG. Ghrelin enhances appetite and increases food intake in humans. J Clin Endocrinol Metab (2001) 86:5992. doi: 10.1210/jcem.86.12.8111 11739476

[103] Im DS. FFA4 (GPR120) as a fatty acid sensor involved in appetite control, insulin sensitivity and inflammation regulation. Mol aspects Med (2018) 64:92–108. doi: 10.1016/j.mam.2017.09.001 28887275

[104] Croze ML Guillaume A Ethier M Fergusson G Tremblay C Campbell SA. Combined deletion of free fatty-acid receptors 1 and 4 minimally impacts glucose homeostasis in mice. Endocrinology (2021) 162:bqab002. doi: 10.1210/endocr/bqab002 33543237

[105] Cintra DE Ropelle ER Moraes JC Pauli JR Morari J Souza CT. Unsaturated fatty acids revert diet-induced hypothalamic inflammation in obesity. PloS One (2012) 7:e30571. doi: 10.1371/journal.pone.0030571 22279596 PMC3261210

[106] Dragano NRV Solon C Ramalho AF de Moura RF Razolli DS Christiansen E. Polyunsaturated fatty acid receptors, GPR40 and GPR120, are expressed in the hypothalamus and control energy homeostasis and inflammation. J Neuroinflamm (2017) 14:91. doi: 10.1186/s12974-017-0869-7 PMC5405534 28446241

[107] Auguste S Fisette A Fernandes MF Hryhorczuk C Poitout V Alquier T. Central agonism of GPR120 acutely inhibits food intake and food reward and chronically suppresses anxiety-like behavior in mice. Int J Neuropsychopharmacol (2016) 19:pyw014. doi: 10.1093/ijnp/pyw014 26888796 PMC4966276

[108] Al Mahri S Malik SS Al Ibrahim M Haji E Dairi G Mohammad S. Free fatty acid receptors (FFARs) in adipose: Physiological role and therapeutic outlook. Cells (2022) 11:750. doi: 10.3390/cells11040750 35203397 PMC8870169

[109] Oh DY Talukdar S Bae EJ Imamura T Morinaga H Fan W. GPR120 is an omega-3 fatty acid receptor mediating potent anti-inflammatory and insulin-sensitizing effects. Cell (2010) 142:687–98. doi: 10.1016/j.cell.2010.07.041 PMC2956412 20813258

[110] Hilgendorf KI Johnson CT Mezger A Rice SL Norris AM Demeter J. Omega-3 fatty acids activate ciliary FFAR4 to control adipogenesis. Cell (2019) 179:1289–1305 e1221. doi: 10.1016/j.cell.2019.11.005 31761534 PMC7332222

[111] Gotoh C Hong YH Iga T Hishikawa D Suzuki Y Song SH. The regulation of adipogenesis through GPR120. Biochem Biophys Res Commun (2007) 354:591–7. doi: 10.1016/j.bbrc.2007.01.028 17250804

[112] Hidalgo MA Carretta MD Burgos RA. Long chain fatty acids as modulators of immune cells function: Contribution of FFA1 and FFA4 receptors. Front Physiol (2021) 12:668330. doi: 10.3389/fphys.2021.668330 34276398 PMC8280355

[113] Hasan AU Ohmori K Hashimoto T Kamitori K Yamaguchi F Noma T. GPR120 in adipocytes has differential roles in the production of pro-inflammatory adipocytokines. Biochem Biophys Res Commun (2017) 486:76–82. doi: 10.1016/j.bbrc.2017.03.001 28263744

[114] Paschoal VA Walenta E Talukdar S Pessentheiner AR Osborn O Hah N. Positive reinforcing mechanisms between GPR120 and PPARγ modulate insulin sensitivity. Cell Metab (2020) 31:1173–1188.e1175. doi: 10.1016/j.cmet.2020.04.020 32413335 PMC7337476

[115] Oh DY Walenta E. Omega-3 fatty acids and FFAR4. Front Endocrinol (2014) 5:115. doi: 10.3389/fendo.2014.00115 PMC4100060 25076939

[116] Quesada-López T Cereijo R Turatsinze JV Planavila A Cairó M Gavaldà-Navarro A. The lipid sensor GPR120 promotes brown fat activation and FGF21 release from adipocytes. Nat Commun (2016) 7:13479. doi: 10.1038/ncomms13479 27853148 PMC5118546

[117] Quesada-López T Gavaldà-Navarro A Morón-Ros S Campderrós L Iglesias R Giralt M. GPR120 controls neonatal brown adipose tissue thermogenic induction. Am J Physiol Endocrinol Metab (2019) 317:E742–50. doi: 10.1152/ajpendo.00081.2019 31361546

[118] Schilperoort M van Dam AD Hoeke G Shabalina IG Okolo A Hanyaloglu AC. The GPR120 agonist TUG-891 promotes metabolic health by stimulating mitochondrial respiration in brown fat. EMBO Mol Med (2018) 10:e8047. doi: 10.15252/emmm.201708047 29343498 PMC5840546

[119] Du YQ Sha XY Cheng J Wang J Lin JY An WT. Endogenous lipid-GPR120 signaling modulates pancreatic islet homeostasis to different extents. Diabetes (2022) 71:1454–71. doi: 10.2337/db21-0794 35472681

[120] Egerod KL Engelstoft MS Lund ML Grunddal KV Zhao M Barir-Jensen D. Transcriptional and functional characterization of the G protein-coupled receptor repertoire of gastric somatostatin cells. Endocrinology (2015) 156:3909–23. doi: 10.1210/en.2015-1388 26181106

[121] Stone VM Dhayal S Brocklehurst KJ Lenaghan C Sörhede Winzell M Hammar M. GPR120 (FFAR4) is preferentially expressed in pancreatic delta cells and regulates somatostatin secretion from murine islets of langerhans. Diabetologia (2014) 57:1182–91. doi: 10.1007/s00125-014-3213-0 PMC4018485 24663807

[122] Zhao YF Li XC Liang XY Zhao YY Xie R Zhang LJ. GPR120 regulates pancreatic polypeptide secretion from Male mouse islets *via* PLC-mediated calcium mobilization. Endocrinology (2020) 161:bqaa157. doi: 10.1210/endocr/bqaa157 32877513

[123] McNelis JC Lee YS Mayoral R van der Kant R Johnson AM Wollam J. GPR43 potentiates β-cell function in obesity. Diabetes (2015) 64:3203–17. doi: 10.2337/db14-1938 PMC4542437 26023106

[124] Villa SR Priyadarshini M Fuller MH Bhardwaj T Brodsky MR Angueira AR. Loss of free fatty acid receptor 2 leads to impaired islet mass and beta cell survival. Sci Rep (2016) 6:28159. doi: 10.1038/srep28159 27324831 PMC4914960

[125] Veprik A Laufer D Weiss S Rubins N Walker MD. GPR41 modulates insulin secretion and gene expression in pancreatic β-cells and modifies metabolic homeostasis in fed and fasting states. FASEB J Off Publ Fed Am Societies Exp Biol (2016) 30:3860–9. doi: 10.1096/fj.201500030R 27550964

[126] Tang C Ahmed K Gille A Lu S Gröne HJ Tunaru S. Loss of FFA2 and FFA3 increases insulin secretion and improves glucose tolerance in type 2 diabetes. Nat Med (2015) 21:173–7. doi: 10.1038/nm.3779 25581519

[127] Tolhurst G Heffron H Lam YS Parker HE Habib AM Diakogiannaki E. Short-chain fatty acids stimulate glucagon-like peptide-1 secretion *via* the G-protein-coupled receptor FFAR2. Diabetes (2012) 61:364–71. doi: 10.2337/db11-1019 PMC3266401 22190648

[128] Park BO Kim SH Kong GY Kim DH Kwon MS Lee SU. Selective novel inverse agonists for human GPR43 augment GLP-1 secretion. Eur J Pharmacol (2016) 771:1–9. doi: 10.1016/j.ejphar.2015.12.010 26683635

[129] Nøhr MK Pedersen MH Gille A Egerod KL Engelstoft MS Husted AS. GPR41/FFAR3 and GPR43/FFAR2 as cosensors for short-chain fatty acids in enteroendocrine cells vs FFAR3 in enteric neurons and FFAR2 in enteric leukocytes. Endocrinology (2013) 154:3552–64. doi: 10.1210/en.2013-1142 23885020

[130] Kimura I Inoue D Hirano K Tsujimoto G. The SCFA receptor GPR43 and energy metabolism. Front Endocrinol (2014) 5:85. doi: 10.3389/fendo.2014.00085 PMC4046487 24926285

[131] Hong YH Nishimura Y Hishikawa D Tsuzuki H Miyahara H Gotoh C. Acetate and propionate short chain fatty acids stimulate adipogenesis *via* GPCR43. Endocrinology (2005) 146:5092–9. doi: 10.1210/en.2005-0545 16123168

[132] Kimura I Ozawa K Inoue D Imamura T Kimura K Maeda T. The gut microbiota suppresses insulin-mediated fat accumulation *via* the short-chain fatty acid receptor GPR43. Nat Commun (2013) 4:1829. doi: 10.1038/ncomms2852 23652017 PMC3674247

[133] Bjursell M Admyre T Göransson M Marley AE Smith DM Oscarsson J. Improved glucose control and reduced body fat mass in free fatty acid receptor 2-deficient mice fed a high-fat diet. American journal of physiology. Endocrinol Metab (2011) 300:E211–220. doi: 10.1152/ajpendo.00229.2010 20959533

[134] Hu J Kyrou I Tan BK Dimitriadis GK Ramanjaneya M Tripathi G. Short-chain fatty acid acetate stimulates adipogenesis and mitochondrial biogenesis *via* GPR43 in brown adipocytes. Endocrinology (2016) 157:1881–94. doi: 10.1210/en.2015-1944 26990063

[135] Dewulf EM Ge Q Bindels LB Sohet FM Cani PD Brichard SM. Evaluation of the relationship between GPR43 and adiposity in human. Nutr Metab (2013) 10:11. doi: 10.1186/1743-7075-10-11 PMC3577645 23327542

[136] Xiong Y Miyamoto N Shibata K Valasek MA Motoike T Kedzierski RM. Short-chain fatty acids stimulate leptin production in adipocytes through the G protein-coupled receptor GPR41. Proc Natl Acad Sci United States America (2004) 101:1045–50. doi: 10.1073/pnas.2637002100 PMC327148 14722361

[137] Zaibi MS Stocker CJ O'Dowd J Davies A Bellahcene M Cawthorne MA. Roles of GPR41 and GPR43 in leptin secretory responses of murine adipocytes to short chain fatty acids. FEBS Lett (2010) 584:2381–6. doi: 10.1016/j.febslet.2010.04.027 20399779

[138] Bellahcene M O'Dowd JF Wargent ET Zaibi MS Hislop DC Ngala RA. Male Mice that lack the G-protein-coupled receptor GPR41 have low energy expenditure and increased body fat content. Br J Nutr (2013) 109:1755–64. doi: 10.1017/s0007114512003923 23110765

[139] Altun I Yan X Ussar S. Immune cell regulation of white adipose progenitor cell fate. Front Endocrinol (2022) 13:859044. doi: 10.3389/fendo.2022.859044 PMC9001836 35422761

[140] Blaszczak AM Jalilvand A Hsueh WA. Adipocytes, innate immunity and obesity: A mini-review. Front Immunol (2021) 12:650768. doi: 10.3389/fimmu.2021.650768 34248937 PMC8264354

[141] Huang C Du W Ni Y Lan G Shi G. The effect of short-chain fatty acids on M2 macrophages polarization *in vitro* and *in vivo* . Clin Exp Immunol (2022) 207:53–64. doi: 10.1093/cei/uxab028 35020860 PMC8802183

[142] Nakajima A Nakatani A Hasegawa S Irie J Ozawa K Tsujimoto G. The short chain fatty acid receptor GPR43 regulates inflammatory signals in adipose tissue M2-type macrophages. PloS One (2017) 12:e0179696. doi: 10.1371/journal.pone.0179696 28692672 PMC5503175

[143] Cong J Zhou P Zhang R. Intestinal microbiota-derived short chain fatty acids in host health and disease. Nutrients (2022) 14:1977. doi: 10.3390/nu14091977 35565943 PMC9105144

[144] Koh A De Vadder F Kovatcheva-Datchary P Bäckhed F. From dietary fiber to host physiology: Short-chain fatty acids as key bacterial metabolites. Cell (2016) 165:1332–45. doi: 10.1016/j.cell.2016.05.041 27259147

[145] Ackerson T Amberg A Atzrodt J Arabeyre C Defossa E Dorau M. Mechanistic investigations of the liver toxicity of the free fatty acid receptor 1 agonist fasiglifam (TAK875) and its primary metabolites. J Biochem Mol Toxicol (2019) 33:e22345. doi: 10.1002/jbt.22345 31066974

[146] Ghislain J Poitout V. Targeting lipid GPCRs to treat type 2 diabetes mellitus - progress and challenges. Nat Rev Endocrinol (2021) 17:162–75. doi: 10.1038/s41574-020-00459-w 33495605

[147] Sun P Wang T Zhou Y Liu H Jiang H Zhu W. DC260126: a small-molecule antagonist of GPR40 that protects against pancreatic β-cells dysfunction in db/db mice. PloS One (2013) 8:e66744. doi: 10.1371/journal.pone.0066744 23776696 PMC3679087

[148] Wu J Sun P Zhang X Liu H Jiang H Zhu W. Inhibition of GPR40 protects MIN6 β cells from palmitate-induced ER stress and apoptosis. J Cell Biochem (2012) 113:1152–8. doi: 10.1002/jcb.23450 22275065

[149] Takeuchi M Hirasawa A Hara T Kimura I Hirano T Suzuki T. FFA1-selective agonistic activity based on docking simulation using FFA1 and GPR120 homology models. Br J Pharmacol (2013) 168:1570–83. doi: 10.1111/j.1476-5381.2012.02052.x PMC3605867 22639973

[150] McCloskey AG Miskelly MG Flatt PR McKillop AM. Pharmacological potential of novel agonists for FFAR4 on islet and enteroendocrine cell function and glucose homeostasis. Eur J Pharm Sci Off J Eur Fed Pharm Sci (2020) 142:105104. doi: 10.1016/j.ejps.2019.105104 31669388

[151] Namour F Galien R Van Kaem T van der Aa A Vanhoutte F Beetens J. Safety, pharmacokinetics and pharmacodynamics of GLPG0974, a potent and selective FFA2 antagonist, in healthy male subjects. Br J Clin Pharmacol (2016) 82:139–48. doi: 10.1111/bcp.12900 PMC4917808 26852904

[152] Miyamoto J Kasubuchi M Nakajima A Kimura I. Anti-inflammatory and insulin-sensitizing effects of free fatty acid receptors. Handb Exp Pharmacol (2017) 236:221–31. doi: 10.1007/164_2016_47 27873088

[153] Cai B Zhang J Zhang M Li L Feng W An Z. Micro-inflammation characterized by disturbed Treg/Teff balance with increasing sIL-2R in patients with type 2 diabetes. Exp Clin Endocrinol Diabetes Off journal German Soc Endocrinol [and] German Diabetes Assoc (2013) 121:214–9. doi: 10.1055/s-0033-1333687 23595796

[154] Rodriguez-Pacheco F Garcia-Serrano S Garcia-Escobar E Gutierrez-Repiso C Garcia-Arnes J Valdes S. Effects of obesity/fatty acids on the expression of GPR120. Mol Nutr Food Res (2014) 58:1852–60. doi: 10.1002/mnfr.201300666 24913719

